# The role of Roux-en-Y hepaticojejunostomy for the management of biliary complications after living donor liver transplantation

**DOI:** 10.1186/s12893-023-02052-0

**Published:** 2023-06-17

**Authors:** Tzu-Cheng Wen, Chia-En Hsieh, Ya-Lan Hsu, Kuo-Hua Lin, Yu-Ju Hung, Yao-Li Chen

**Affiliations:** 1 Department of General Surgery, Changhua Christian Hospital, Changhua, Taiwan; 2 Department of Surgery, Liver Transplant Center, Chung Shan Medical University Hospital, No. 110, Sec. 1, Jianguo N. Rd, South Dist, Taichung City, 402306 Taiwan, ROC; 3 School of Medicine, Chung Shan Medical University, Taichung, Taiwan, ROC

**Keywords:** Living donor liver transplantation, Roux-en-Y hepaticojejunostomy, Biliary complication

## Abstract

**Introduction:**

Post living donor liver transplantation (LDLT) biliary complications can be troublesome over the post-operative course of patients, especially those with recurrent cholangitis or choledocholithiasis. Thus, in this study, we aimed to evaluate the risks and benefits of Roux-en-Y hepaticojejunostomy (RYHJ) performed after LDLT as a last option to deal with post-LDLT biliary complications.

**Methods:**

Retrospectively, of the 594 adult LDLTs performed in a single medical center in Changhua, Taiwan from July 2005 to September 2021, 22 patients underwent post-LDLT RYHJ. Indications for RYHJ included choledocholithiasis formation with bile duct stricture, previous intervention failure, and other factors. Restenosis was defined if further intervention was needed to treat biliary complications after RYHJ was performed. Thereafter, patients were categorized into success group (*n* = 15) and restenosis group (*n* = 4).

**Results:**

The overall success rate of RYHJ in the management of post-LDLT biliary complications was 78.9% (15/19). Mean follow-up time was 33.4 months. As per our findings, four patients experienced recurrence after RYHJ (21.2%), and mean recurrence time was 12.5 months. Three cases were recorded as hospital mortality (13.6%). Outcome and risk analysis presented no significant differences between the two groups. A higher risk of recurrence tended to be related to patients with ABO incompatible (ABOi).

**Conclusion:**

RYHJ served well as either a rescue but definite procedure for recurrent biliary complications or a safe and effective solution to biliary complications after LDLT. A higher risk of recurrence tended to be related to patients with ABOi; however, further research would be needed.

## Introduction

Living donor liver transplantation (LDLT) had gained popularity over the recent decades and proved to be as curative as deceased donor liver transplantation (DDLT) [1]. However, the presence of biliary complications remain as an insurmountable difficulty in terms of LDLT, especially those receiving a right lobe liver graft [2].

Post-LDLT biliary complication rate can vary between centers and countries, ranging from 9.6% to 19% [3–6]. Efforts taken to minimize post-LDLT biliary complications had evolved over the years. Prevention of local ischemia of biliary tract and preceding bile leakage might contribute to reducing post-operative risk of biliary anastomosis stricture [7, 8]. Intraoperatively, technical modifications of choledochocholedochostomy helped improved the stricture rate. A theoretically higher risk of biliary tract ischemia was suggested of an end-to-end anastomosis as it added fragility and an impaired perfusion at the distal end of the bile duct [8, 9].

However, with the nature of graft and possibility of rejection, a certain percentage of patients were still found to be at high risk of post-LDLT biliary complications [7, 8]. The necessity of managing such conditions was challenging yet inevitable. With the progress of techniques and equipment over the recent years, endoscopic maneuvers had largely replaced the role of surgical intervention while dealing with biliary complications after LDLT [10]. Percutaneous transhepatic drainage (PTCD) also gained popularity for it served as a second-line management for patients who were treated with endoscopy yet deemed unsuccessful or those who were not suitable to receive an endoscopic procedure.

The above endoscopic techniques could help out almost 80% of the patients suffering from post-LDLT biliary complications [11], with varying success rates from 53 to 88% [12]. Some patients may experience procedure failure probably due to a sharp angle of the biliary tract or severe stenosis. Intrahepatic duct (IHD) stones formation might also lead to recurrent cholangitis. A rescue percutaneous treatment could temporarily relieve obstructive jaundice or cholangitis and prevent progression of infection. However, drainage tube may cause inconvenient to the patient and may not thoroughly solve bile duct stricture. Under such circumstances, re-anastomosis of the biliary-enteric route seemed to be necessary. A Roux-en-Y hepaticojejunostomy (RYHJ) is the choice of surgical method [13–16]. The aim of this study is to evaluate the safety and recurrence risk factors of RYHJ performed after LDLT as a last resort of dealing with post-LDLT biliary complications.

## Materials and methods

**Fig. 1:**
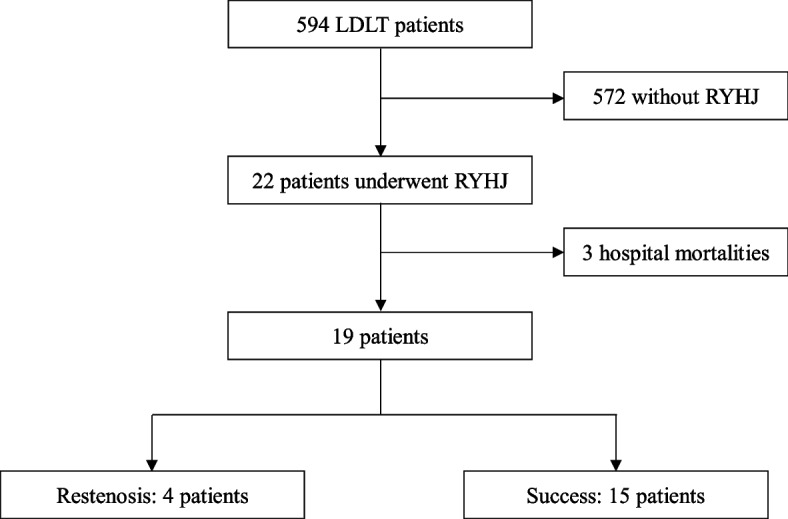
Inclusion and exclusion criteria for patient underwent Roux-en-Y hepaticojejunostomy after living donor liver transplantation. LDLT: Living donor liver transplantation; RYHJ: Roux-en-Y hepaticojejunostomy

Between July 2005 and September 2021, 594 patients who had underwent LDLT were retrospectively recruited from Changhua Christian Hospital in Taiwan. Patients between 18 and 85 years old with a living donor right lobe graft and subsequently RYHJ were enrolled into this study. Exclusion criteria include patients whose ages were under 18 years old or over 85 years old at the time of operation. Among the 22 patients identified, 3 were excluded due to hospital mortality. In total, 19 patients met the inclusion criteria and were enrolled in this study for outcome and risk factor analysis (Fig. 1). Restenosis was defined if further intervention was needed to treat biliary complications after RYHJ was performed. Patients were then categorized into the success group (*n* = 15) and restenosis group (*n* = 4). This study was approved by the Institutional Review Board of Changhua Christian Hospital, Changhua, Taiwan.

### surgical technique

The recipient surgery technique was mentioned in previous published literature [9].

Post-LDLT RYHJ was performed by a single surgeon. A right subcostal incision was made with a midline extension to the xiphoid. The adhesive bowel and stomach were lysed from the liver in order to locate and expose the liver hilum well. During hilar adhesion lysis, we paid extra attention toward the dissection of the hepatic artery (HA). After carefully identifying the HA and the common bile duct (CBD), cavitational ultrasonic surgical aspirator was applied to dissect the liver parenchyma around the graft right IHD. The previous right IHD – common hepatic duct anastomosis was identified and resected. Roux limb was designed from proximal jejunum. The anastomosis of hepaticojejunostomy was done in a fashion of continuous suture of the posterior wall and interrupt suture of the anterior wall. Moreover, 6–0 monofilament, nonabsorbable polypropylene sutures were used for the anastomosis. We enveloped the Glissonean sheath of the graft right IHD to the anastomosis to prevent early bile leak. While performing the anastomosis, a nasogastric tube or T-tube was often used as an intra-ductal stent. The choice of the size of tube depends on the diameter of graft right IHD measured intraoperatively. Drainage tubes were placed over Morison pouch and left subphrenic space.

### Data collection

Patient demographic data was retrospectively gathered from each patient. The age, gender and Model for End-Stage Liver Disease (MELD) score were recorded during history taking on admission for operation or chart record review. Operational-related data were extracted from operative records. The presence of rejection was defined as post-operative administration of bolus corticosteroid. The diagnosis of bile leak was made with biloma formation noted on contrast computed tomography or contrast leakage noted on cholangiogram or cholangioscopy. Intrahepatic duct stone was based on the finding of computed tomography or filling defects noted on cholangiogram or cholangioscopy [17]. Patients were followed up until March 2022. Recurrence was defined if further intervention was needed to treat biliary complications after RYHJ was performed.

All of the above data were reviewed and confirmed by two of the researchers of this literature.

### Statistical analysis

Quantitative data are expressed as means and standard deviations, whereas categorical variables are summarized as numbers and percentages. The Pearson chi-square (Fisher) test and Mann–Whitney U test were used to examine differences in demographic and clinical characteristics within the two groups. A *p*-value lower than 0.05 was statistically significant in our analysis. All statistical analyses were performed using IBM SPSS Statistics, version 22.0.

## Results

### Patient characteristics

From July 2005 and September 2021, 594 patients underwent LDLT in our center. The mean age of the patients receiving RYHJ was 52.53 ± 6.04 years old. Clinical and operative characteristics of patients of LDLT who underwent subsequent RYHJ were listed individually in Table 1. Summative data analysis was listed in Table 2.

**Table 1:** Individual demographic data and clinical features of patients of LDLT who underwent RYHJ

	Recipient age (years)	Gender	MELD score	Cold ischemia time (min)	Warm ischemia time (min)	Anhepatic phase time(min)	RL-LDLT operative time (min)	RL-LDLT blood loss (ml)	RYHJ operative time (min)	RYHJ blood loss (ml)	Time from RL-LDLT to RYHJ (month)
1	51	Male	6	114	1	45	390	1500	270	250	49.5
2	47	Male	13	155	1	29	420	700	250	250	48
3	50	Male	13	145	38	49	540	3800	195	300	60.5
4	60	Male	19	29	23	88	390	4400	325	650	30
5	56	Male	16	64	22	100	370	6000	290	1750	18
6	50	Male	22	95	25	32	360	7000	195	150	46.5
7	58	Male	13	113	34	49	330	3650	275	300	19.5
8	58	Female	27	103	20	27	390	1400	245	250	31
9	47	Male	9	83	15	18	315	800	265	350	16
10	59	Female	11	90	25	26	360	1100	220	100	27.5
11	65	Male	7	66	21	22	345	600	325	300	14.5
12	60	Female	11	55	24	31	365	3000	240	650	11.5
13	47	Male	25	92	18	28	180	7000	255	250	24
14	53	Male	17	66	24	25	435	2300	240	100	10
15	49	Male	9	86	14	15	250	1000	215	100	10.5
16	50	Male	10	55	17	26	260	1500	530	500	5
17	45	Male	20	35	18	15	370	3000	225	300	12
18	52	Male	9	44	17	64	335	8000	270	550	6
19	45	Male	28	64	17	19	320	3500	365	1500	2.5
20	71	Male	13	114	15	28	310	5000	445	1700	3.5
21	46	Male	7	114	15	28	390	800	180	100	0.3
22	39	Female	24	23	15	18	280	2200	280	7000	1.5

	Bile leak	Bile duct anastomosis orifice no	Type of bile duct anastomosis	T-Tube placement	Hepatic artery complications	IHD stone formation	ABO incompatibility	Rejection	Survival	Hospital Mortality
1	Yes	2	D-D	No	No	Yes	No	Yes	Yes	No
2	Yes	2	D-D	No	No	Yes	No	No	No	No
3	Yes	1	D-D	Yes	No	Yes	No	Yes	Yes	No
4	No	1	D-G	Yes	No	Yes	No	No	Yes	No
5	Yes	3	D-G	No	No	Yes	No	No	No	No
6	No	1	D-G	No	No	Yes	No	Yes	Yes	No
7	No	1	D-G	No	No	Yes	No	No	No	No
8	No	2	D-G	No	No	Yes	Yes	Yes	Yes	No
9	No	1	D-G	No	Yes	Yes	No	No	Yes	No
10	No	2	D-G	No	No	Yes	Yes	No	Yes	No
11	No	2	D-G	No	No	No	No	No	Yes	No
12	No	1	D-G	No	No	Yes	No	No	No	No
13	Yes	1	D-G	Yes	Yes	Yes	No	No	No	No
14	No	1	D-G	No	Yes	Yes	No	No	Yes	No
15	No	1	D-G	No	No	No	No	Yes	Yes	No
16	No	1	D-D	Yes	No	Yes	Yes	Yes	No	Yes
17	No	1	D-D	No	No	No	Yes	No	Yes	No
18	Yes	1	D-D	Yes	No	No	No	Yes	Yes	No
19	Yes	1	D-D	No	No	No	No	No	Yes	No
20	Yes	1	D-D	No	No	No	No	No	No	Yes
21	Yes	2	D-D	No	No	No	No	No	Yes	No
22	Yes	2	D-D	No	No	No	No	Yes	No	Yes

*RL-LDLT* Right lobe living donor liver transplantation, *RYHJ* Roux-en-Y hepaticojejunostomy, *SD* Standard deviation, *MELD* Model for end-stage liver disease, *D-G* Duct-to-Glissonean sheath anastomosis, *D-D* Duct-to-duct anastomosis, *IHD* Intrahepatic duct

**Table 2:** Summative demographic data and clinical features of patients of LDLT who underwent RYHJ

	Median (range)	
Recipient age (years)	50.5 (39–71)	
MELD score	13 (6–28)	
Cold ischemia time (min)	84.5 (23–155)	
Warm ischemia time (min)	18 (1–38)	
Anhepatic phase time(min)	28 (15–100)	
Transplantation operative time (min)	360 (180–540)	
Transplantation blood loss (ml)	2650 (600–8000)	
Hepaticojejunostomy operative time (min)	260 (180–530)	
Hepaticojejunostomy blood loss (ml)	300 (100–7000)	
Time from RL-LDLT to RYHJ (month)	23.5 (0–58)	
	No.	(%)
Gender		
Male	18	81.8
Female	4	18.2
Bile leak		
Yes	12	54.5
No	10	45.5
No. of bile duct orifice		
1	14	63.6
2	7	31.8
3	1	4.5
Type of bile duct anastomosis		
D-G	12	54.5
D-D	10	45.5
T-tube placement		
Yes	5	22.7
No	17	77.3
Hepatic artery complications		
Yes	3	13.6
No	19	86.4
IHD stone		
Yes	14	63.6
No	8	36.4
ABO incompatibility		
Yes	4	18.2
No	18	81.8
Rejection		
Yes	8	36.4
No	14	63.6
Survival		
Alive	14	63.6
Death	8	36.4

*LDLT* Living donor liver transplantation, *RYHJ* Roux-en-Y hepaticojejunostomy, *SD* Standard deviation, *MELD* Model for End-Stage Liver Disease, *D-G* Duct-to-Glissonean sheath anastomosis, *D-D* Duct-to-duct anastomosis, *IHD* Intrahepatic duct

### Recurrence risk and outcome analysis

Nineteen out of the 22 patients from the patients of LDLT who underwent subsequent RYHJ and didn’t experience hospital mortality were enrolled for recurrence risk and outcome analysis. Overall, the success rate of RYHJ in managing post-LDLT biliary complications was 78.9% (15/19). Mean follow up time was 33.4 months. Four patients experienced recurrence after RYHJ (21.2%), and mean recurrence time was 12.5 months. Comparing the group of patients who suffered from biliary complication recurrence and those who did not, no variable was proved to be a significant risk factor regarding recurrence after RYHJ. Comparisons of demographic data and clinical features of between patients with and without restenosis were listed in Table 3.

**Table 3:** Comparisons of demographic data and clinical features of patients of RL-LDLT who underwent RYHJ

Demographic and clinical features	Success group (*n* = 15)	Restenosis group (*n* = 4)	*p*
	*Median (IQR)*	*Median (IQR)*	
Recipient age (years)	50.0 (47.0–57.0)	54.5 (50.0–58.3)	0.596
MELD score	13.0 (10.0–19.5)	10.0 (8.3–15.0)	0.411
Cold ischemia time (min)	66.0 (59.5–104.0)	96.5 (88.3–105.8)	0.307
Warm ischemia time (min)	21.0 (17.0–24.0)	17.5 (11.5–21.3)	0.469
Anhepatic phase time(min)	29.0 (23.5–49.0)	26.5 (24.0–31.5)	0.469
Transplantation blood loss (ml)	3500.0 (1650.0–5200.0)	1250.0 (1025.0–1425.0)	0.124
Transplantation operative time (min)	365.0 (332.5–390)	375.0 (348.8–390.0)	0.961
Hepaticojejunostomy blood loss (ml)	300.0 (200.0–600.0)	250.0 (212.5–275.0)	0.411
Hepaticojejunostomy operative time (min)	250.0 (220.0–282.5)	255.0 (238.8–266.3)	0.961
Post Transplantation length (month)	14.5 (10.3–27.0)	29.3 (24.6–35.6)	0.185
	*n (%)*	*n (%)*	
Gender			0.097
Male	14 (93.3)	2 (50.0)	
Female	1 (6.7)	2 (50.0)	
Bile leak			0.603
Yes	7 (46.7)	1 (25.0)	
No	8 (53.3)	3 (75.0)	
Bile duct anastomosis orifice			0.108
1	11 (73.3)	1 (25.0)	
2	3 (20.0)	3 (75.0)	
3	1 (6.7)	0 (0)	
Type of bile duct anastomosis			1.000
D-G	9 (60.0)	3 (75.0)	
D-D	6 (40.0)	1 (25.0)	
T-Tube placement			0.530
Yes	4 (26.7)	0 (0)	
No	11 (73.3)	4 (100)	
Hepatic artery complications			0.530
Yes	2 (13.3)	1 (25.0)	
No	13 (86.7)	3 (75.0)	
IHD stone			0.255
Yes	9 (60.0)	4 (100)	
No	6 (40.0)	0 (0)	
ABO incompatibility			0.097
Yes	1 (6.7)	2 (50.0)	
No	14 (93.3)	2 (50.0)	
Rejection			0.557
Yes	4 (26.7)	2 (50.0)	
No	11 (73.3)	2 (50.0)	
Survival			0.530
Alive	10 (66.7)	4 (100.0)	
Death	5 (33.3)	0(0)	

*RL-LDLT* Right lobe living donor liver transplantation, *RYHJ* Roux-en-Y hepaticojejunostomy, *IQR* Interquartile range, *MELD* Model for end-stage liver disease, *D-G* Duct-to-Glissonean sheath anastomosis, *D-D* Duct-to-duct anastomosis, *IHD* Intrahepatic duct

Three cases were recorded as hospital mortality, where in one of them suffered from HA injury intraoperatively during RYHJ; soon complicated with early bile leak, the patient expired due to subsequent sepsis. The other two patients had severe infections after endoscopic interventions and underwent RYHJ, yet multiple organ failure developed for malicious systemic infection and both of them expired.

During long-term follow-up, five patients expired in the non-recurrence group, and all other patients were currently under regular outpatient follow up. Among them, two patients suffered from hepatocellular carcinoma recurrence; one died due to metastatic obstructive pneumonia; one had aortic stenosis and underwent aortic valve replacement, yet the patient expired due to post-operative myocardial rupture; and one passed away for esophageal varicose bleeding with hepatorenal syndrome. The average follow-up time for those expired was 20.7 months.

## Discussions

Post LDLT biliary complications is an issue commonly discussed yet still not fully understood, especially those with a right lobe liver graft [17–19]. Even with advanced equipment and improved techniques, a certain percentage of patients still might not be thoroughly managed with non-operative interventions. The high failure rate of LDLT patients may be due to small-caliber bile duct anastomosis and multiple and more complex fibrotic anastomoses; it can also be attributed to liver graft hypertrophy [17]. Under certain circumstances, RYHJ seemed to be the last resort in terms of dealing with post-LDLT biliary complications [8]. Hepaticojejunostomy for biliary tract obstruction following liver transplantation was recommended for patient with endoscopic intervention failure. Also, the surgical procedure provided a more effective way in the management of cases with complicated biliary complications, such as necrosis of the bile duct, IHD stones, or extensive biliary leakage, since its procedural steps help established a thorough view of the major biliary system and biliary concrements can be extracted by flushing all major bile ducts under direct visualization [16].

Our study focused on these patients who suffered from either non-operative intervention failure or other clinical conditions not suitable for endoscopic or radiologic interventions (i.e., choledocholithiasis, acute bleeding, septic shock, etc.) The biliary-enteric anastomosis may provide an eternal solution to difficult shaped CBD or non-anastomotic biliary strictures [8, 10]. According to previous analysis, the overall success rate of our study was 78.9% (15/19). Four patients experienced recurrence after RYHJ (21.2%), and mean recurrence time was 12.5 months. Another center reported their experience with 16% of complication rate and 11% of restenosis rate [14]. Chok. et al. published a series of studies on RYHJ managements after LDLT, proving a promising overall success rate with 76% [16, 20, 21] and thus making surgical managements of post-LDLT a salvaging last resort. They also suggested that side-to-side RYHJ could be a more suitable choice over side-to-end anastomosis, for the previous could reduce the chance of HA injury and allow a better chance for post-operative endoscopic procedure [20]. In post-orthotopic liver transplantation RYHJ, a high-volume single center provided their experience with a mean survival rate of 70% and a 5-year survival rate of 68%, proving it as a promising rescue procedure for post-liver transplantation biliary obstruction not resolved by endoscopic procedures [13].

Three patients were recorded as hospital mortality in our analysis. One of the patients suffered from HA insufficiency postoperatively, even after stent placement. The patient developed intrahepatic biloma and subsequent liver failure. We recognized HA insufficiency may be a troublesome condition to deal with in post-LDLT RYHJ. Two of the patients underwent the surgery after intra-abdominal infection developed due to multiple failure episodes of endoscopy. We hence would like to suggest that RYHJ should be performed right after non-surgical intervention has failed. Mortality rate of post-LDLT RYHJ tended to be higher if the patient was presented with pre-operative infection.

**Fig. 2:**
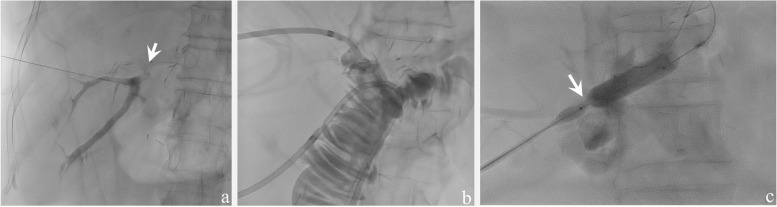
Percutaneous transhepatic cholangiography of patients presented with restenosis after post-LDLT RYHJ. **a**. Contrast introduced through percutaneous transhepatic route revealed none had reached the Roux limb. Arrow: Contrast introduced through percutaneous transhepatic route revealed a near total obstruction of the biliary-enteric tract. **b**. Pigtail drainage tubes were placed via the guidewire under fluorescence guidance, functioned as stents for the biliary-enteric anastomosis and transhepatic biliary drainage at the same time. **c**. The percutaneous transhepatic cholangiographic three-episode balloon dilatation. Arrow: The balloon passing through the stenosis part of the biliary tract

Recurrence of biliary complications after RYHJ seemed to be frustrating yet inevitable (Fig. 2a). Recurrent IHD stones or restenosis of the more upstream biliary tree (e.g., right posterior branch) was observed in these patients even after RYHJ was performed. Although non-significantly, we noted a relative higher percentage of ABOi in patients with biliary complication recurrence after RYHJ. In current clinical practices, a standardized desensitization protocol with anit-CD20 antibody (rituximab) and plasma exchange had proved its ability of providing a comparable outcome for adult LDLT recipients receiving an ABOi graft [8, 22–25]. Despite that, the incidence of biliary complications still remained higher than ABO-compatible patients [23], ranging from 12.4% [26] to 50% [27]. RYHJ may not be a definite treatment for ABOi patient who suffered from post-LDLT biliary complications, for ABO antigens were found on bile duct epithelium and might cause continuous intrahepatic bile duct injury due to immune response activation [8, 28]. As we review the pre-operative antigen titer before plasma exchange, those with recurrence aren’t higher. During follow-up, the concentration of tacrolimus and antigen titers is all within acceptable range according to chart records. This may suggest the possibility of episodes of subclinical inflammations in these patients due to persisted biliary epithelial injury and thus the failure of RYHJ. A relative higher percentage of female patients was noted in the restenosis group. Previous literatures rarely reported gender as a significant risk factor for biliary complications in liver transplantation [29, 30]. Verdonk et al. reported that female donor/male recipient pair was a significant risk factor post-transplantation anastomosis stricture. However, sex mismatch had only been reported as a risk of chronic rejection and failure of the graft [31], and our data discrepancy might be caused by a small number of case and data bias.

Among the four patients with symptomatic recurrence after RYHJ, we arranged PTC drainage (Fig. 2b) and subsequently a three-episode balloon dilatation protocol for most of them (Fig. 2c). The average time of protocol initiation was 47.8 months after RYHJ, immediately after biliary stricture was diagnosed. To date, all of the patients who received this protocol were currently free from symptomatic biliary complications.

This study has limitations. Although our case number was comparable to other literatures, it is still a rather small size cohort. Selection restriction was inevitable for there’s a significant difference in operative indications between early and late RYHJ patients. This may indicate a fundamental distinction between the two groups of patients. Analysis bias could possibly be presented considering such circumstances.

## Conclusion

RYHJ served well as either a rescue but definite procedure for recurrent biliary complications or a safe and effective solution to early biliary complications after LDLT. We reported that none of the factor was proved to be a recurrence risk factor regarding the failure of RYHJ after LDLT for biliary stenosis managements. Although non-significantly, a relatively higher percentage of recurrence seemed to be related to patients with ABOi.

## Data Availability

The data that support the findings of this study are available from Changhua Christian Hospital, but restrictions apply to the availability of these data, which were used under license for the current study, and so are not publicly available. Data are however available from the authors upon reasonable request and with permission of Changhua Christian Hospital.

## Abbreviations

LDLT: Living donor liver transplantation
RYHJ: Roux-en-Y hepaticojejunostomy
ABOi: ABO-incompatible
DDLT: Deceased donor liver transplantation
PTCD: Percutaneous transhepatic drainage
IHD: Intrahepatic duct
HA: Hepatic artery
CBD: Common bile duct
MELD: Model for End-Stage Liver Disease
